# Preventive screening for intracranial aneurysms

**DOI:** 10.1177/17474930211024584

**Published:** 2021-06-17

**Authors:** Gabriel JE Rinkel, Ynte M Ruigrok

**Affiliations:** Department of Neurology and Neurosurgery, UMC Utrecht Brain Center, University Medical Center Utrecht, Utrecht, The Netherlands

**Keywords:** Intracranial aneurysm, subarachnoid hemorrhage, screening, familial

## Abstract

**Background:**

Subarachnoid hemorrhage from rupture of an intracranial aneurysm (aneurysmal subarachnoid hemorrhage) is a devastating subset of stroke. Since brain damage from the initial hemorrhage is a major cause for the poor outcome after aneurysmal subarachnoid hemorrhage, prevention of aneurysmal subarachnoid hemorrhage has the highest potential to prevent poor outcome from aneurysmal subarachnoid hemorrhage.

**Aim:**

In this review, we describe the groups at high risk of aneurysmal subarachnoid hemorrhage who may benefit from preventive screening for unruptured intracranial aneurysms followed by preventive treatment of unruptured intracranial aneurysms found. Furthermore, we describe the advantages and disadvantages of screening and advise how to perform counseling on screening.

**Summary of review:**

Modeling studies show that persons with two or more affected first-degree relatives with aneurysmal subarachnoid hemorrhage and patients with autosomal dominant polycystic kidney disease (ADPKD) are candidates for screening for unruptured intracranial aneurysms. One modeling study also suggests that persons with only one affected first-degree relative with aneurysmal subarachnoid hemorrhage are also likely candidates for screening. Another group who may benefit from screening are persons ≥35 years who smoke(d) and are hypertensive, given their high lifetime risk of aneurysmal subarachnoid hemorrhage of up to 7%, but the prevalence of unruptured intracranial aneurysms in such persons and the efficiency and cost-effectiveness of screening in this group are not yet known. The ultimate goal of screening is to increase the number of quality years of life of the screening candidates, and therefore the benefits but also many downsides of screening –such as risk of incidental findings, very small unruptured intracranial aneurysms that require regular follow-up, preventive treatment with inherent risk of complications and anxiety – should be discussed with the candidate so that an informed decision can be made before intracranial vessels are imaged.

**Conclusions:**

Several groups of persons who may benefit from screening have been identified, but since these constitute only a minority of all aneurysmal subarachnoid hemorrhage patients, additional high-risk groups still need to be identified. Further research is also needed to identify persons at low or high risk of aneurysmal development and rupture within the groups identified thus far to improve the efficiency of screening. Moreover, if new medical treatment strategies that can reduce the risk of rupture of unruptured intracranial aneurysm become available, the groups of persons who may benefit from screening could increase considerably.

## Background

Aneurysmal subarachnoid hemorrhage (ASAH) from rupture of an intracranial aneurysm (IA) is a devastating subset of stroke. The mean age at which it occurs is around 50 years, which is much lower than for the more common types of stroke. Another difference with more common types of stroke is that it occurs more often in women than in men.^1^ One third of the patients die in the initial days to weeks after ASAH.^2^ Of those who survive these initial weeks, a considerable proportion remains dependent on help from others, and even those who have a so-called good outcome, defined as being able to take care of own affairs, often have cognitive complaints or deficits that preclude resuming all pre-morbid activities. All in all, only 5% of patients can take up their pre-morbid life without any restrictions or complaints.^3^

Since brain damage from the initial hemorrhage is a major cause for the poor outcome after ASAH,^4^ prevention of ASAH has the highest potential to prevent poor outcome from ASAH. IAs are hardly ever symptomatic before they rupture, and therefore screening is the only way to detect IAs before rupture, and to install preventive treatment. The purpose of screening is, however, not to detect IAs, or to prevent ASAH, but to improve the number of life years in good quality of life, and persons who seek advice on screening should be properly informed about the benefits, but also the risks of screening so that they can make an informed decision whether or not to undergo screening.

For medical interventions, the best available evidence are randomized clinical trials, or meta-analyses thereof. Such trials have never been done for screening of high-risk groups of ASAH, and the chance that such trials will ever be performed is small given the large number of persons that should participate in such trials, the decades of follow-up needed for such a trial, and last but not least the unlikeliness that a sufficient number of potential participants will agree to participate. Persons with a familial preponderance of ASAH are an important group of potential screening candidates, or trial participants, but if someone already has seen close relatives dying or being impaired from ASAH, the likelihood that someone agrees in no screening but regular follow-up is probably small. In the absence of randomized trials, modeling studies are a good alternative and therefore evidence that screening is useful therefore comes only from such studies.^5^ For such a modeling approach, data are needed on chances of finding an unruptured intracranial aneurysm (UIA) and of rupture of the aneurysm in the particular subgroup of persons, and on life expectancy, risk of complications from preventive aneurysm treatment, and level of anxiety in both the screened and non-screened persons. Modeling studies can also inform on the cost-effectiveness of screening strategies, a factor to be considered since screening and preventive treatment of UIAs come with costs.

In this review, we describe the high-risk groups for ASAH, the evidence for screening in these high-risk groups, the disadvantages of screening, and advise on how to counsel persons who seek advice on screening.

## High-risk groups

### Familial intracranial aneurysms

Persons with a positive family history for ASAH are the largest group of persons who may benefit from screening and preventive aneurysm treatment, since around 10% of all ASAH patients have a one or more relatives who also had an ASAH.^6,7^

### Persons with two or more first-degree relatives with ASAH

For persons with two or more affected first-degree relatives, the estimated lifetime risk of ASAH can be higher than 20%,^8^ depending on the type of relation and presence or absence of other risk factors (Table 1). The life-time risk is higher for siblings than for parents or children of affected patients,^9^ and increases in persons who smoke or have hypertension.^10^

**Table 1.** Chance of finding an aneurysm and estimated life time risk of aneurysmal subarachnoid hemorrhage (ASAH) according to number of affected first-degree relatives

Number affected first-degree relatives	Chance of finding aneurysm at screening	Estimated life time risk of ASAH^a^
0	3.2% (95% CI 1.9–5.2)^48^	0.5%^1^
1	4% (95% CI 2.6–5.8)^19^	3–4%^8,49–50^
2 or more	11% (95% CI 9–14)^11^	Up to 20%^8^

95% CI: 95% confidence interval.

a The estimates are based on the incidence rates and life expectancy numbers; accordingly no uncertainty levels can be calculated.

The chance of finding an UIA at screening of a person with two or more affected relatives is around 10% at initial screening, and around 5–7% at follow-up screening with five-year intervals (Table 1).^11^ This chance is independent from the age of the screened person after the third decade of age. This means that the upper age limit of screening does not depend on the chance of finding an UIA, but on the balance between remaining life expectancy and risk of complications of preventive aneurysm treatment. Although women have a slightly higher chance of having an UIA than men if the affected relative is a woman,^12^ this difference is too small to take sex into account when counseling on screening.

The risk of rupture of familial aneurysms has long been considered very high. This was based on a relative risk of 17 found in a study comparing the risk of rupture found in the Familial Intracranial Aneurysm study with that of comparable aneurysms in the International Study of Unruptured Intracranial Aneurysm.^13^ Besides the indirect comparison between two cohorts of patients, other limitations of the study are the strict selection of patients with familial aneurysms: apart from having a positive family history, relatives should also have had a history of smoking, hypertension or both for inclusion in the study.^13^ Moreover, this high relative risk was based on only two instances of ASAH. Recent data from a single center study comparing familial and non-familial aneurysm indeed showed an increased rupture risk for familial aneurysm, but to a more modest level with a tripled risk.^14^

Although familial aneurysms tend to rupture at younger age than sporadic aneurysms,^15^ there is no good concordance of age at time of rupture within families. Thus, age of the screened person found to have an aneurysm does not help to discriminate high- from low-risk aneurysms within families. Similarly, there is no good concordance in aneurysm size at rupture within familial IAs,^16^ which indicates that size of a ruptured IA in a family member should not influence the management of a familial UIA in a relative.

The high life-time risk of ASAH, the high chance of finding an UIA at screening and the increased risk of rupture of familial aneurysms all suggest that persons with two or more affected first-degree relatives are good candidates for screening. Indeed, two modeling studies have shown that screening is cost-effective in these circumstances with the optimal strategy being to screen every 5–7 years between ages 20 and 70–80 years of age.^17,18^

### Persons with one first-degree relative with ASAH

Persons with only one first-degree relative with ASAH have long been considered no good candidates for screening, based on a modeling study that showed an increased life expectancy but a reduced number of quality-adjusted life years (QALYs) after screening and preventive treatment of UIAs.^19^ This model was developed over 25 years ago, and since then additional factors that may influence the outcome of this model have become available. First, newer data have shown that the risk of ASAH in such persons is higher than presumed when the model was developed, with an estimated lifetime risk of 3–4%.^20^ This risk is considerably higher than the risk for persons with no affected relatives, which is around 0.5%, based on the incidence of ASAH in the general population (Table 1).^1^ Second, the risk of complications of preventive aneurysm occlusion has decreased over the last decades, not only through the advent of endovascular treatment but also through a lower risk of complications from surgical treatment.^21^ These data suggest that the balance on benefits and risks of screening might have changed in favor of screening. Indeed, a single more recent modeling study using current data showed that screening such persons can be cost-effective, with a threshold of €20.000 per QALY. The strategy with highest net health benefit was screening such persons twice, at ages 40 and 55.^22^ More frequent screening still increases health benefits, but at high additional costs. Some uncertainty remains regarding the cost-effectiveness because the ‘probability’ that screening is cost-effective for most strategies is around 50% and not above the 70% that is required to confirm with certainty that it is cost-effective. However, based on these data, we feel persons should be informed on the increased risk of ASAH, and counseling on screening should be offered.

### Persons with first-degree relatives with UIA

Most studies thus far have focused on relatives with ASAH or have not discriminated between relatives with ASAH and relatives with UIAs. There are no good data on prevalence of UIA or risk of ASAH for persons who have one or more relatives with UIA, but no relatives with ASAH. Thus, for such persons the efficiency of screening is unknown, and we tend to discourage such persons from screening. For persons with one first-degree relative with ASAH and one or more relatives with UIA, we advise screening every five years, despite lacking evidence for this particular subset of persons.

### Autosomal polycystic kidney disease (ADPKD)

ADPKD is an autosomal dominant hereditary disorder in which patients develop cysts in kidneys and sometimes liver.^23^ ADPKD patients have a high risk of hypertension and cardiovascular disease including UIA and ASAH.^24–26^ The estimated prevalence of UIA is 10%,^24^ which is higher in case of a positive family history for hemorrhagic stroke (no distinction made in type of hemorrhage, so not specific for ASAH alone) or UIA with a risk ratio of 2.3 (95% CI 1.6–3.4).^24^ ADPKD is most often caused by mutations in the genes PKD1 (in 78% of families) or PKD2 (in 15% of families).^27^ Up to 6% of ADPKD patients might die of ASAH.^25^ In a recent prospective series of 495 ADPKD patients, ASAH incidence was 2.0/1000 patient-years,^26^ thus 20 times higher than in the general population. Although ASAH is common in patients with ADPKD, only 1% of all ASAH cases is attributable to ADPKD^28^ because ADPKD is relatively rare with a prevalence of 1/1000 individuals.^29^ ASAH occurs at a younger age in patients with ADPKD (median age 42.8 years) than in patients without ADPKD (median age 52.8 years).^28^ In addition, ASAH occurs from smaller aneurysms in patients with ADPKD than in patients without ADPKD (6.00 vs 8.00 mm).^28^ Two recent modeling studies show that preventive screening for UIA in patients with ADPKD is cost-effective, regardless of the presence of a positive family history.^26,30^ Repeated screening every five years is advised after a negative initial study,^30^ because of the 10% risk of a new ASAH in the first decade after the initial episode.^31^

### Other conditions associated with UIA and ASAH

Other conditions associated with UIA and ASAH include the rare, genetic connective tissue disorders type IV Ehlers-Danlos syndrome (vascular subtype),^32–34^ Marfan syndrome^32,35^ and Loeys-Dietz syndrome,^32,36–37^ but the series describing UIA and ASAH in these disorders are small. UIA and ASAH have also been associated with other (non-connective tissue) conditions fibromuscular dysplasia (FMD),^38,39^ coarctatio aortae,^40^ and bicuspid aortic valve,^40^ but also for these associations this evidence is weak because based on small patient series. Therefore, reliable estimates on occurrence of UIAs and ASAH cannot be made. Moreover, little is known about the rupture risk of aneurysms, or the complication risks of preventive treatment in patients with these specific conditions. This makes evidence-based advice not possible. Some experts from international consortia screening have, however, expressed the opinion that screening may be considered for type IV Ehlers-Danlos,^34^ Loeys-Dietz syndrome,^37^ and FMD.^39^

### Potential additional high-risk groups eligible for screening

A group who may also benefit from screening are persons ≥35 years of age who smoke(d) and are hypertensive given their high lifetime risk of aSAH of up to 7%.^41^ Together with the group of patients with a positive family history, this high risk group constitutes 30% of all ASAH patients.^41^ Therefore, a third of all ASAH cases could potentially be prevented with an optimal preventive screening strategy in these two groups of high-risk individuals. However, the prevalence of UIAs in persons ≥35 years who smoke(d) and are hypertensive, and thus the efficiency and cost-effectiveness of screening in this group are not yet known.

## Counseling on screening

The purpose of counseling is providing the screening candidate the information on benefits and risks of screening she/he needs to make an informed decision on whether or not to undergo screening. Important is that the person feels sufficiently informed to make a sound decision. As the ultimate goal of screening is to increase the number of quality years of life of the screening candidates, the risks and benefits of screening should be weighed, before intracranial vessels are imaged (Table 2). First, it is important that screening is performed only in patients without significant co-morbidity as in case of co-morbidity the advantages of screening may not sufficiently outweigh its disadvantages. Magnetic-resonance angiography (MRA) can be used to screen for UIAs and has the advantages over CT angiography (CTA) that no contrast injection is needed and there is no radiation exposure. In case of contra-indications for MRA, such as claustrophobia or previously neurosurgical clipping treatment with the clips causing extensive artefact on MRA,^42^ CTA can be performed instead. The purpose of screening, i.e. preventing ASAH and consequently increasing the number of quality years of life in the future, should be discussed. Herewith it is important to realize that with repeated screening and preventive treatment of UIAs identified not all episodes of ASAH can be prevented. In rare instances, aneurysms can develop and rupture within the regular screening interval of five years.^43^ Moreover, the optimal age for screening, the chance of an ASAH and the yield of screening for the screening candidate need to be outlined. The need of repeated screening should also be mentioned. Possible disadvantages of screening should be discussed as well. These include the risk of complications in case of preventive treatment of an UIA found at screening^21^ and the risk of finding a small UIA that is still too small to be treated at that time and must be followed up for possible growth with follow-up imaging.^44^ If the screening candidate has children, he/she should be informed that if on screening an UIA is identified, his/her children also become eligible for screening. It should also be mentioned that there is a chance of incidental findings other than aneurysms, including an asymptomatic brain infarcts, meningioma, cavernoma or a hypophyseal adenoma.^45^ Last, the implications for driving and flying licenses and life insurance, which may differ by country, should be discussed. At the end of the consultation, it is up to the patient to make an informed decision whether or not to undergo screening. Sometimes, a follow-up consultation proves necessary to give a patient an opportunity to reflect on the decision or for further discussion or information. Finally, all patients, even those who decide not to be screened, should be advised to stop smoking and have their blood pressure checked regularly because also in persons with familial risk or ADPKD, smoking and hypertension increase the risks for both aneurysm development and ASAH.^44,46^

**Table 2.** Items that should be discussed when counseling candidates on screening for unruptured intracranial aneurysms

Items
Purpose of screening
Chance of finding an UIA during initial and follow-up screening
Risk of ASAH
Types of preventive treatment of UIAs and inherent risks
Treatment of risk factors hypertension and smoking
Ages to start and stop screening
Chance of finding UIAs which are too small for preventive treatment and require regular follow-up imaging
Screening decreases the risk of ASAH, but does not completely rules out this risk – there remains a small risk
Consequences of screening outcome for children of screening candidate
Implications for driving and flying licences
Implications for life insurance
Risk of incidental findings
Give candidates time to think

UIA: Unruptured intracranial aneurysm; ASAH: aneurysmal subarachnoid hemorrhage.

## Conclusions and future perspectives

Based on the data presented, persons with two or more affected first-degree relatives with ASAH and patients with ADPKD are candidates for screening for IA. Current data also suggest that screening increases the number of quality adjusted life years at acceptable costs in persons with only one first-degree relative with ASAH. We feel such persons should also be informed about the higher risk they have for ASAH and about the possibility of counseling for screening.

Given the downsides there are with screening, persons should be counseled before imaging is ordered. The purpose of counseling is to present all relevant data to persons in such a way that they can make a truly informed decision on whether or not they want to undergo screening. For the physician who counsels the screening candidate, the ultimate interest of the encounter is not whether or not to have convinced the screening candidate for the most appropriate decision according to the statistical model, but a patient who feels to have been able to make an informed decision.

Because the groups of persons who may benefit from screening constitute only a minority of all ASAH patients, additional high risk groups still need to be identified. Moreover, within the groups of persons identified to benefit from screening, the current screening strategies for these groups are still inefficient. Many persons undergo several cycles of screening without ever having an aneurysm, and some persons still have ASAH despite screening. Future research on genetics, including on rare genetic variants in familial aneurysms and genetic risk scores predicting UIA or ASAH, and on anatomical risk factors such as configuration of the circle of Willis, may identify persons at low or high risk of aneurysmal development and rupture within the groups identified thus far, which may render screening more efficient. Similarly, data on absolute risks of UIA or ASAH according to age, number of affected relatives, and environmental risk factors as smoking and hypertension may further discriminate low- from high-risk persons within the group of persons with a positive family history. Current treatment strategies for preventive aneurysm occlusion carry considerable risks, which reduce the benefit of screening. If new treatment strategies to reduce the risk of rupture of UIA, such as medical treatment with antihypertensive drugs and aspirin,^47^ become available, the groups of persons who may benefit from screening could increase considerably.
